# Augmented Reality as a Tool to Guide PSI Placement in Pelvic Tumor Resections

**DOI:** 10.3390/s21237824

**Published:** 2021-11-24

**Authors:** Mónica García-Sevilla, Rafael Moreta-Martinez, David García-Mato, Alicia Pose-Diez-de-la-Lastra, Rubén Pérez-Mañanes, José Antonio Calvo-Haro, Javier Pascau

**Affiliations:** 1 Departamento de Bioingeniería e Ingeniería Aeroespacial, Universidad Carlos III de Madrid, 28911 Leganés, Spain; mongarci@pa.uc3m.es (M.G.-S.); rmoreta@pa.uc3m.es (R.M.-M.); dgmato@ing.uc3m.es (D.G.-M.); apose@ing.uc3m.es (A.P.-D.-d.-l.-L.); 2 Instituto de Investigación Sanitaria Gregorio Marañón, 28007 Madrid, Spain; rubenperez.phd@gmail.com (R.P.-M.); calvoharo@yahoo.es (J.A.C.-H.); 3 Servicio de Cirugía Ortopédica y Traumatología, Hospital General Universitario Gregorio Marañón, 28007 Madrid, Spain

**Keywords:** augmented reality, patient-specific instruments, pelvic tumor resection, 3D printing

## Abstract

Patient-specific instruments (PSIs) have become a valuable tool for osteotomy guidance in complex surgical scenarios such as pelvic tumor resection. They provide similar accuracy to surgical navigation systems but are generally more convenient and faster. However, their correct placement can become challenging in some anatomical regions, and it cannot be verified objectively during the intervention. Incorrect installations can result in high deviations from the planned osteotomy, increasing the risk of positive resection margins. In this work, we propose to use augmented reality (AR) to guide and verify PSIs placement. We designed an experiment to assess the accuracy provided by the system using a smartphone and the HoloLens 2 and compared the results with the conventional freehand method. The results showed significant differences, where AR guidance prevented high osteotomy deviations, reducing maximal deviation of 54.03 mm for freehand placements to less than 5 mm with AR guidance. The experiment was performed in two versions of a plastic three-dimensional (3D) printed phantom, one including a silicone layer to simulate tissue, providing more realism. We also studied how differences in shape and location of PSIs affect their accuracy, concluding that those with smaller sizes and a homogeneous target surface are more prone to errors. Our study presents promising results that prove AR’s potential to overcome the present limitations of PSIs conveniently and effectively.

## 1. Introduction

The treatment of malignant tumors in the pelvis presents a complex scenario for surgeons, since extensive resections are required in many cases, with the risk of damaging vital structures. In these interventions, accuracy is crucial to minimize local recurrence and maximize the functionality of the patient’s limb. However, when performing the tumor excision following the conventional freehand approach, the probability of obtaining adequate margins is only 52% (95% CI: 37–67) [1].

In these complex procedures, computer-assisted surgery (CAS) has become a valuable tool for translating preoperative planning to the operating room [2,3,4,5,6,7]. Navigation systems can track surgical instruments and display their position in relation to the patient’s anatomy on preoperative images (computed tomography or magnetic resonance). Surgeons can benefit from this technique to guide the excision and achieve adequate resection margins.

The use of patient-specific instruments (PSI) for surgical guidance is also widely extended [8,9,10,11]. These three-dimensional (3D) printed tools are designed to fit in a particular region of the patient’s bone and guide the osteotomies. In contrast to surgical navigation, PSIs do not provide real-time visual feedback, but they have reported similar accuracy [12] and allow surgeons to focus on the surgical field rather than looking at the navigation display. They are also easier to use, more convenient, and faster. However, their accuracy is subject to the correctness of their placement, which highly depends on PSI shape and bone morphology. Finding the correct position in smooth and homogeneous regions becomes challenging, and tissue covering the bone can also hamper their installation. Moreover, the placement cannot be verified objectively during the intervention [12], increasing the risk of high osteotomy deviations resulting from incorrect positioning. In the pelvis, the ilium presents a large and homogenous shape, and thus the installation of PSIs for tumor resection is more prone to errors [8,13,14]. Therefore, additional tools assisting PSI placement would be of great value in these surgical scenarios.

Another technology that is gaining popularity in surgical procedures, including orthopedic surgery, is augmented reality (AR) [15,16]. It displays computer-generated elements which augment and enrich the visualization of the physical world. Devices such as smartphones or head-mounted displays are commonly used for this purpose. Inside the operating room, AR mainly focuses on visualizing the tumor and other anatomical structures [17,18,19], enhancing guidance during resections. The virtual models can be displayed in accordance with the patient’s anatomy by performing a registration step, which is either calculated manually [20], using tracking systems [21,22], through advanced algorithms for surface registration [23], or by markers detected with pattern recognition techniques [24]. A recent study presented a solution consisting of a 3D printed pattern that can be detected by an RGB (Red-Green-Blue) camera [17]. This pattern is attached to a PSI, whose position in the patient is known, and therefore the virtual elements are displayed in place. Several studies have proved this solution evaluating feasibility, convenience, and accuracy in different surgical scenarios [17,18,25].

Studies focused on estimating the accuracy provided by these technologies are performed in a living subject [26], a cadaver [8,12,13,27], or a phantom [1,10,18]. Although phantoms provide a less realistic environment, they present several advantages apart from avoiding patient risks. They are readily available and can provide more consistent results as they offer a controlled environment where the anatomy is maintained. It is more difficult to compare results between cases in real surgical settings as the anatomy and other factors change and need to be considered for the analysis. Depending on the application, phantoms can be designed replicating the anatomical structures or the composition of human tissue in more or less detail. In addition, thanks to 3D printing, patient-specific phantoms can be generated more easily for personalized measurements [28]. Several studies have used phantoms to report the accuracy results from CAS, PSIs, or AR in pelvic tumor resections [1,10]. These phantoms consist of a replica of the bone, usually made of plastic. However, when evaluating PSIs placement, as the smoothness and homogeneity of the bone’s surface and the presence of tissue are essential factors, the results provided by these phantoms may not be realistic enough.

In this study, we propose to use AR as a tool to guide PSIs placement in pelvic tumor resections, overlying virtual models of the PSIs on the patient to indicate their planned position to surgeons. These models are displayed in place thanks to a 3D printed AR marker attached to a PSI. We assess the accuracy provided by the system on two phantoms, a conventional one consisting only of the plastic pelvic bone and a realistic phantom including a layer of silicone simulating tissue. Six pairs of PSIs were designed representing six different scenarios for tumor resection in the ilium. Four users placed the PSIs in both phantoms following three different methodologies: freehand, AR with a smartphone, and AR with the HoloLens 2. The results prove the value of using AR to minimize placement errors, showing how simulated tissue in the phantom complicates placement and underlying the importance of the area in which PSIs are installed.

## 2. Materials and Methods

**Figure 1:**
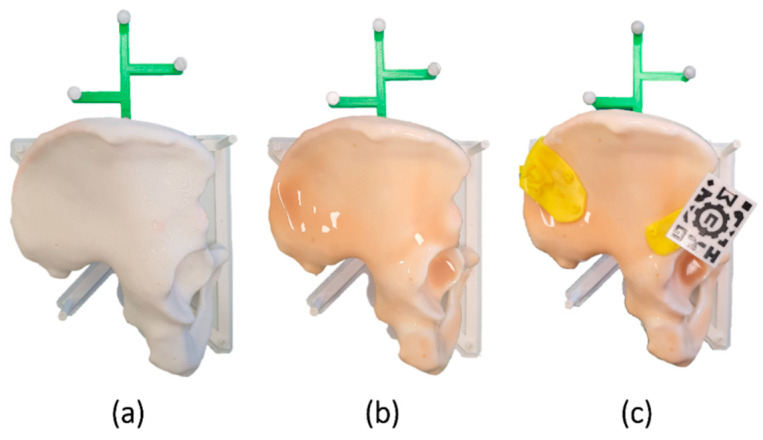
(**a**) Conventional bone phantom and (**b**) realistic phantom including a silicone layer. (**c**) patient-specific instruments (PSIs) and augmented reality (AR) marker placed on the phantom.

We designed a phantom based on the computed tomography (CT) image of a patient (Figure 1a). The bone model was extracted with the 3D Slicer platform [29] through a segmentation process using thresholding and divided with a sagittal plane to keep only the right hemipelvis. Using Meshmixer software (Autodesk, Inc., San Rafael, CA, USA), we designed a base for the phantom to place it in a similar position to that found in an actual surgery (“floppy lateral” [30]). A reference frame was added to the design for navigation. Finally, the bone phantom was 3D printed using the Ultimaker 3 Extended (Ultimaker B.V., Utrecht, The Netherlands) desktop 3D printer in polylactic acid (PLA).

Six scenarios were designed for tumor resections in the ilium (type I according to Enneking and Dunham’s classification [31]). Each scenario was composed of two PSIs indicating the osteotomies, one in the supra-acetabular region (S) and one in the iliac crest (C). The designed PSIs presented different sizes, shapes, and locations inside the target region. All PSIs were created in Meshmixer following the steps described by García-Sevilla et al. [13] and 3D printed in polylactic acid (PLA) (Figure 1c).

Since PSIs are designed based on the bone model, they easily fit into the phantom, adapting to the surface like a puzzle. Only imperfections introduced during printing can hinder their placement. However, in a real scenario, there are other factors to consider that affect placement. As previously mentioned, there is frequently tissue in contact with the bone that has not been completely removed. Additionally, errors in the segmentation process can generate discrepancies between the virtual model and the patient’s bone. Consequently, the surface of the PSIs would no longer match the bone seamlessly. Therefore, to better reproduce that real scenario, a silicone layer (Ecoflex^TM^ 00-10) was overlaid on the phantom, simulating the tissue and adding complexity to the placement (Figure 1b). The silicone was extended across the surface with a brush and cured within 4 h.

An AR marker similar to the one described by Moreta-Martinez et al. [17] was used to display the virtual models overlaid on the phantom (Figure 1c). This marker presented a unique black and white pattern that an RGB camera could recognize. It was 3D printed in PLA with the Ultimaker 3 Extended 3D printer using the dual extruder functionality. We added a prism-shaped pillar to the design. Thus, the marker could be attached to the surgical guides by inserting it in a socket included in the PSIs. The position of the marker in the PSIs and the bone can be known with precision, as this attachment has demonstrated repeatability in previous studies [14].

**Figure 2:**
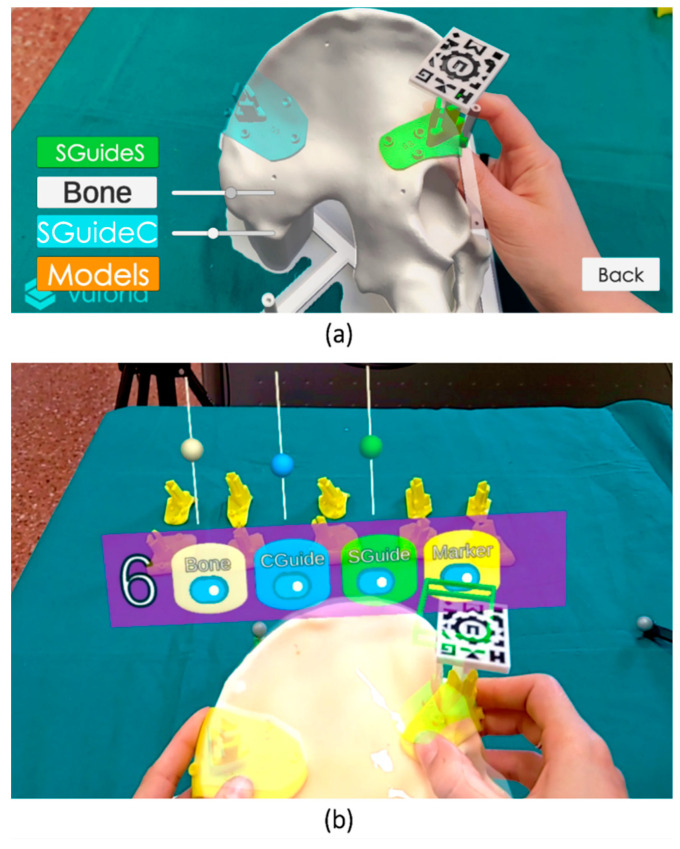
Screenshots of the AR visualization with the (**a**) smartphone and (**b**) HoloLens 2 during PSIs placement. Specific interactive panels of each device are shown, containing visibility and opacity buttons and sliders.

Two software applications were developed to display the virtual models with AR, one to be deployed in a smartphone (Figure 2a) and a similar one for the Microsoft HoloLens 2 device (Figure 2a). Both applications were implemented on Unity platform (version 2019.3), using the Vuforia development kit (Parametric Technology Corporation Inc., Boston, MA, USA) for pattern recognition. Details regarding the development of an AR app for these purposes have been previously described by Moreta-Martinez et al. [32]. The applications included a menu to select the case and buttons to change the visibility and opacity of each model. In the smartphone app, this interaction was done on the screen. For the HoloLens 2, an interactive panel was shown in front of the user. The applications included the visualization of virtual models of the PSIs for that specific case, as well as a model of the bone. The idea was to adjust the placement of S PSIs using the bone model as a reference. Once fixed, the planned position of C PSI would be displayed and used as guidance. The steps followed for AR guidance are therefore the following:

1. Placement of S PSI
2. Placement of AR marker in S PSI
3. Detection of the marker with the AR device and visualization of the virtual models
4. Adjustment of S PSI so that virtual and physical bone models are visually aligned
5. Placement of C PSI so that it overlaps the overlayed virtual C PSI

The Supplementary Material contains a demo video for each device showing the procedure and interaction with the apps. The described steps are included in the videos (Videos S1 and S2). The Supplementary Material also includes a table (Table S1) with details regarding all the software and hardware elements used in our study.

**Figure 3:**
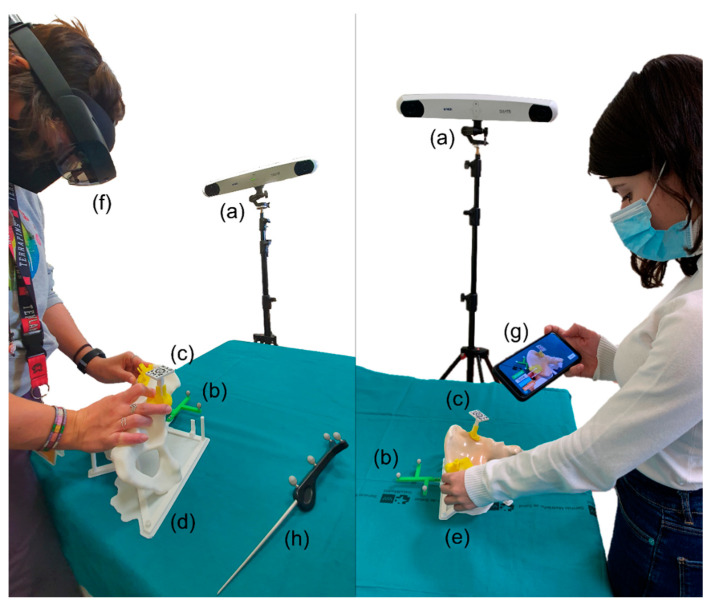
Setup for the assessment using (**left**) the HoloLens 2 and (**right**) the smartphone. The setups include the following elements: (**a**) optical tracker, (**b**) reference frame, (**c**) AR marker placed in PSI, (**d**) conventional, and (**e**) realistic phantom versions, (**f**) HoloLens 2, (**g**) smartphone, and (**h**) pointer.

We designed an experiment to assess the accuracy provided by the system. Four users without clinical background placed the PSIs for each of the six cases following three different methodologies: freehand, AR guidance with a smartphone, and AR (or Mixed Reality) guidance with the HoloLens 2 (Figure 3). In order to replicate the setup we would follow in the operating room, users held the smartphone with one hand and used the other one to place the PSIs. This setup allowed them to move the smartphone freely and change the point of view. As the smartphone can be introduced in a sterile case, the surgeons could hold it in the same way inside the operating room and maintain asepsis. Users repeated the process with the conventional phantom (without silicone) and with the realistic version (with silicone). We wanted to compare the results for each method and detect whether the addition of silicone significantly increased the placement difficulties. We were also interested in analyzing how the different shapes and locations of the PSIs affected the results. Additionally, we measured the time required for each user to place the PSIs for each scenario.

To record the position of the PSI during the experiment, we used the Polaris Spectra (NDI, Waterloo, ON, Canada) optical tracking system and the SlicerIGT [33] module for navigation included in the 3D Slicer software. The reference frame with spherical passive markers included in the bone phantom allowed to track its position (Figure 3). The position of this reference frame with respect to the phantom is defined in the design. However, it was attached to the phantom with screws, so we could expect small differences in rotations or translations from the designed pose. Hence, we performed a registration step to ensure that the target registration errors were minimized. Initial point-based registration was calculated from artificial landmarks recorded on the bone with a tracked pointer. To ensure an accurate registration, the result was then refined with the iterative closest point algorithm (ICP) [34] calculated on points recorded across the surface. The registration was only performed once at the beginning of the experiment and before adding the layer of silicone to ensure an accurate surface registration. This way, the registration was maintained for all cases, methods, and phantom versions. The ICP registration error was below 0.1 mm.

**Figure 4:**
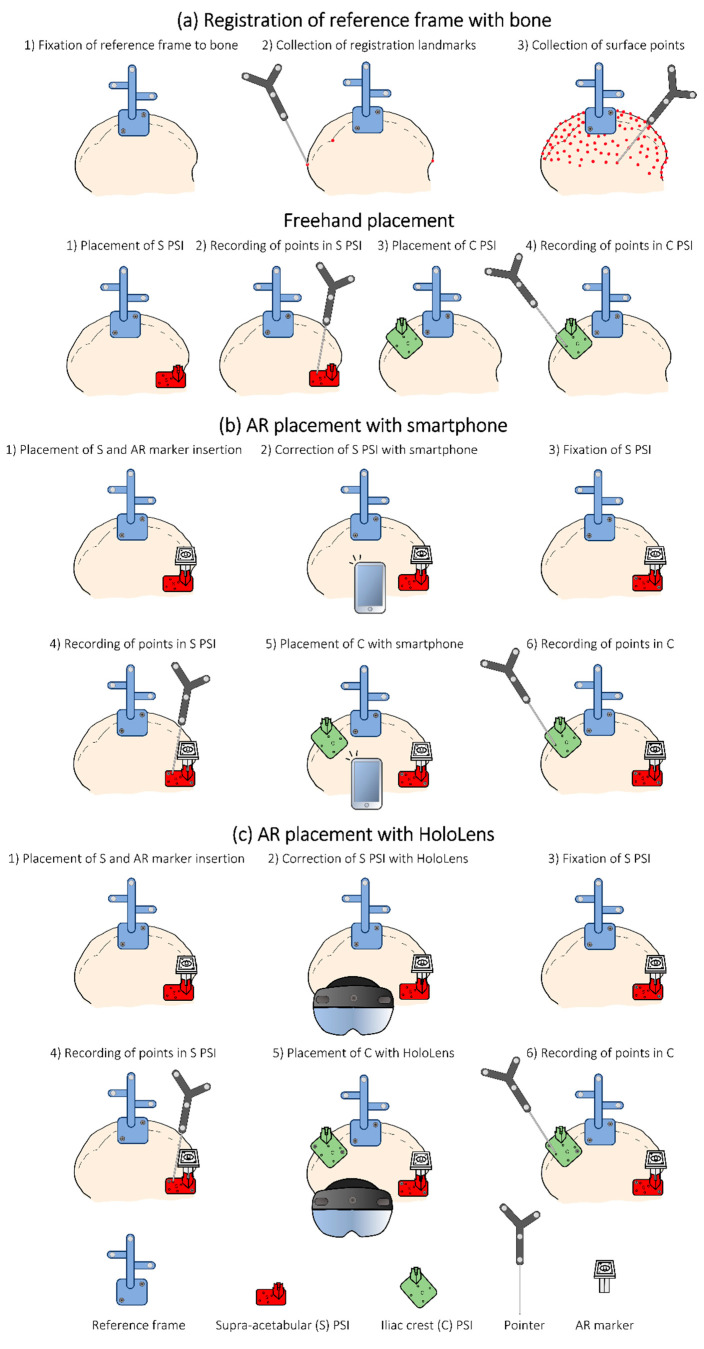
Steps followed during the experiment for registration and to record PSIs placements in each methodology. (**a**) Registration of reference frame with bone, (**b**) AR placement with smartphone, and (**c**) AR placement with HoloLens.

During the experiment, we obtained the position of four pinholes present in every PSI with the pointer. These points were later used to obtain the transformation of the PSI from its planned position to the actual one (planToReal). Figure 4 represents the steps followed in each scenario for the assessment.

**Figure 5:**
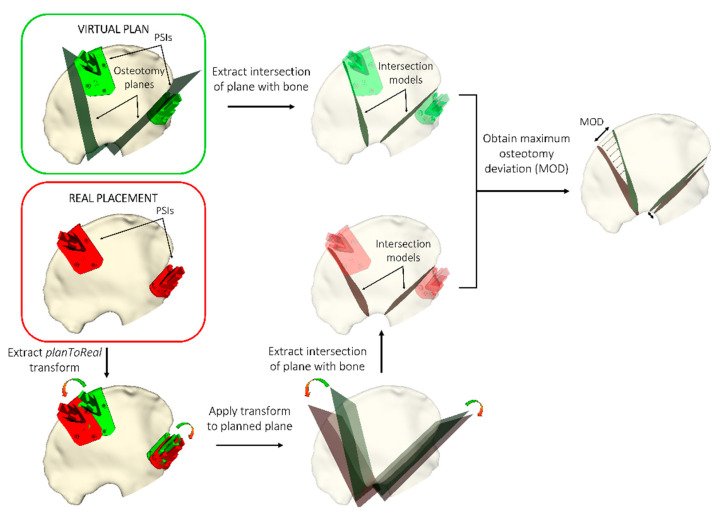
Computation of the maximum osteotomy deviation (MOD) between planned and real osteotomy planes. The real osteotomy is defined by the PSI placement performed by the user.

When using PSIs, we are interested in minimizing the errors or deviations from the planned osteotomy. Hence, we extracted the maximum osteotomy deviation (MOD) for each PSI to measure the system’s accuracy. For that, we first found the intersection of the planned osteotomy planes with the bone in S and C for each case. Then, we applied the corresponding planToReal transform to the planned osteotomy planes and extracted their intersection with the bone, obtaining a 3D model (point cloud). Finally, we computed the distances between every point in the planned intersection model to its closest point from the actual intersection model. The maximum distance obtained for that case was defined as the MOD. These steps are represented graphically in Figure 5.

Finally, we analyzed the statistical difference between the MODs obtained for each method (freehand, AR with smartphone and AR with HoloLens 2), phantom (conventional and realistic) and case, obtaining the median (Mdn) and interquartile range (IQR).

## 3. Results

We computed the MOD for all cases with the conventional and the realistic phantom. Table 1 presents descriptive statistics of these measurements, where the results for each guidance method are presented divided by regions (C and S) and combined.

The results obtained using the smartphone or the HoloLens 2 present median values for MOD below 2 mm in the realistic phantom and below 1 mm in the conventional one. These deviations are below 1.6 mm in 75% of the non-silicone cases and below 2.7 mm in 75% of those with silicone. Without silicone, errors are slightly higher for the C region, while they are lower than S when using silicone. The maximum errors recorded are 3 mm in the conventional phantom and 5 mm in the realistic phantom.

When PSIs are placed freehand, the median value for MODs is 1.70 mm with the conventional phantom and 3.37 mm with the realistic one. The C region presents the highest deviations, with maximum errors of 7.27 mm without silicone and 54.03 mm with silicone. The 75th percentile in C presents an error of 10.02 mm with the silicone phantom, which is considerably higher than the 2.64 mm on the non-silicone one.

We conducted a statistical analysis to identify significant differences between methods, cases, and phantoms. The results for each analysis are presented in the following subsections.

### 3.1. Methods Comparison

A Kruskal–Wallis test showed that the method chosen significantly affects the results, H (2) = 25.80, *p* < 0.001. The freehand method presented higher maximum deviations (Mdn = 2.22, IQR = 1.23–3.63) than using the smartphone (Mdn = 1.20, IQR = 0.85–1.86) or the HoloLens 2 (Mdn = 1.33, IQR = 0.97–2.11). Post-hoc Dunn’s test was used to compare all pairs of methods. The difference between HoloLens and the smartphone was not significant (*p* = 0.47). However, the differences between using the freehand method and using either HoloLens or the smartphone were significant (*p* < 0.001 in both cases). Taken together, the results suggest that placing the PSIs freehand affects MODs, increasing the risk of high errors considerably.

We also recorded the time taken by each user to place the PSIs in every scenario. The Kruskal–Wallis test proved significant differences among methods (H (2) = 10.19, *p* = 0.006). The time required with the freehand method (Mdn = 48.5 s, IQR = 30.50–48.50) was significantly lower than using AR guidance with the smartphone (Mdn = 75, IQR = 68.25–110.25) or HoloLens (Mdn = 78.5, IQR = 62.25–106). However, these time values are negligible compared to the intraoperative surgical time for these procedures.

### 3.2. Cases Comparison

**Figure 6:**
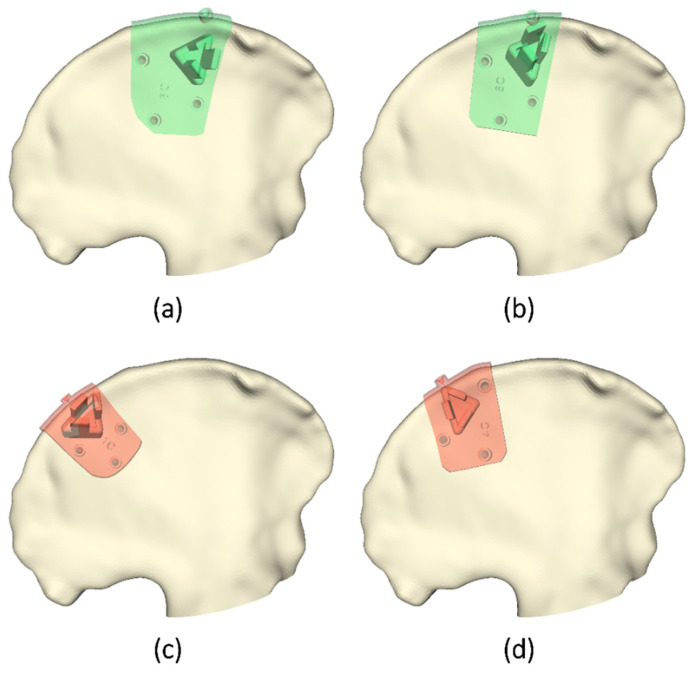
Cases with iliac crest PSIs presenting significant differences with each other. The figures represent the cases with (**a**,**b**) lower and (**c**,**d**) higher errors.

Deviations were also compared among cases for the C and S regions. A Kruskal–Wallis test was calculated, obtaining not statistically significant differences in S, but significant differences in C, with H (5) = 33.50 and *p*-value < 0.001. The Dunn’s test indicated significant differences between cases 1 (Mdn = 2.07, IQR = 1.48–5.00) (Figure 6c) and 4 (Mdn = 1.73, IQR = 1.49–2.58) (Figure 6d) with cases 3 (Mdn = 0.97, IQR = 0.64–1.15) (Figure 6a) and 5 (Mdn = 1.11, 0.73–1.37) (Figure 6b). If we analyze the results from C considering their size and position, we can observe how the highest errors correspond to smaller PSIs placed in smoother regions. In comparison, the best results are obtained for larger PSIs covering less homogeneous regions. The S region is narrower than C, and therefore there are fewer variations in position among PSIs.

### 3.3. Phantoms Comparison

Finally, we compared the results obtained using the realistic phantom with those from the conventional phantom by conducting a Kruskal–Wallis test. Significant differences were obtained for the freehand method (H (1) = 18.07, *p* < 0.001), the smartphone (H (1) = 14.07, *p* < 0.001) and the HoloLens (H (1) = 17.12, *p* < 0.001). Errors were higher in all cases when using the phantom with the layer of silicone.

## 4. Discussion

The correct placement of PSIs in pelvic tumor resections is a critical step that conditions the results obtained in surgery. However, there is no current solution to guide their installation, and surgeons can only rely on their intuition and verify their placement visually. External devices providing guidance and allowing an objective verification can therefore represent a significant improvement in using PSIs, minimizing errors and improving precision.

In this work, we propose the use of AR to guide and verify this placement. Our setup is based on 3D printed AR markers that snap into the PSIs at a certain position. By visualizing the bone model and the relative position between PSIs, surgeons can guide their placement in the patient. We focus our study on the ilium, the region of the pelvis presenting more placement difficulties. Our setup includes two PSIs, one in S and one in C. The AR marker is placed in the S PSI since placement errors in this area are lower due to its small and characteristic shape. The bone model is used to correct S PSI placement. After this adjustment, the planned virtual model of the C PSI is displayed to a guide the placement.

We designed an experiment with four users who placed the PSIs on a phantom following three different methods: freehand, AR with a smartphone, and AR with HoloLens 2. Then, we computed the MOD between the cutting planes defined by the placed PSIs and the cutting planes obtained from the preoperative plan. The results demonstrate that placing PSIs with AR guidance significantly reduces MOD errors. Specifically, it avoids placements far away from their planned position. AR methods presented median values below 2 mm and MODs below 5 mm in all cases, while the freehand method recorded higher median values (1.70 mm and 3.37 mm) and maximum errors of 54 mm. As for the AR methods, both devices presented similar results. Therefore, selecting one over the other is more dependent on the surgeon’s personal preferences and convenience. The smartphone can be easily inserted into a sterile bag or case such as CleanCase (Steridev Inc., Lansing, MI, USA) [18], but it requires the surgeons to hold it, leaving only one hand free. On the other hand, the HoloLens 2 does not require holding, but it can generate discomfort in long-term use and is more expensive.

Our study was performed on two phantoms, a conventional one consisting only of a plastic replica of the bone and a second one including a layer of silicone to simulate tissue and add difficulty to placement. The results obtained show higher errors in all methods when using the realistic phantom. In the case of AR methods, this may be a consequence of a thicker layer of silicone present in some areas, specifically in S. This explains why, with AR methods, the realistic phantom presented higher errors in S, while similar values were maintained for both phantoms in C. Nevertheless, the differences between both phantoms are especially remarkable in the freehand method, where the median error is duplicated, going from 1.70 mm in the conventional phantom to 3.37 mm in the realistic one. This increase can be noted in both regions, S and C. Therefore, the results obtained reinforce the idea that using plastic phantoms without any material simulating tissue makes the placement easier and does not resemble the real scenario correctly.

Although the silicone coating served its purpose by adding difficulty during placement, the phantom’s design can be further improved. When we poured the silicone, the entire surface was covered smoothly. However, in a real setting, the tissue is not evenly distributed. Therefore, future studies could improve the design of the phantom to make it more realistic by adding small fractions of silicone with different thicknesses unevenly distributed.

A tumor model could also be added to the phantom to provide more realistic measurements, as in surgical settings it can be used as a reference for placing the PSIs. The surgeons can notice that the direction of the osteotomy crosses the tumor or is too deviated from it and correct the PSI placement accordingly. However, to simulate this with a phantom, the tumor should be included with enough realism to resemble the surgical scenario, where tumor margins are not clearly defined. Therefore, we believe including a 3D printed tumor would not be realistic enough and could result in lower deviations than in real surgical settings. Additionally, in our study we wanted to measure and compare the deviations of the osteotomies with different methodologies in different scenarios. In order to minimize the possible factors introducing variations in the assessment, the same phantom and reference frame registration was used for all the experiments, and the scenarios were simply modified by changing the target positions of the PSIs. The introduction of a tumor for each case would have implied designing and printing a different phantom for each scenario. Therefore, 3D printing inaccuracies would cause variations among phantoms, and registrations errors would not be the same, modifying the conditions between scenarios. Hence, we consider our setup to be adequate, although it must be noted that the reported deviations in the freehand method could present lower values in a clinical setting. Changes in dimensions and shape of the tumor from the acquisition of the preoperative image to the moment of the intervention are factors that can affect the surgical outcomes when using surgical guidance based on preoperative planning. These modifications impact both surgical navigation and PSIs. This known limitation can only be solved by using intraoperative imaging. However, these changes do not affect the registration step for AR guidance, as it is based on the position of the PSIs in the bone. Only changes in the target bone area can prevent from using PSIs, with or without AR. In that case, the procedure could be performed following the conventional approach without any guidance.

The shape, size and location chosen during PSIs design have shown to be highly relevant factors to consider for minimizing placement errors. In our study, those cases presenting both small size and homogeneous target area exhibited significantly higher errors than those with larger sizes and a more distinct target surface. However, the available target area may be limited depending on the surgical case, leaving no choice but to choose a homogeneous surface. Regarding size, although larger designs are easier to install in the correct location, they are also more invasive [8,35]. Hence, a trade-off between ensuring precision and reducing invasiveness should be found.

Further testing should be performed in a clinical setup to analyze the placement accuracy and validate the system in a real scenario. Nevertheless, using PSIs combined with AR inside the operating room has already been tested in previous studies (for other purposes) [17,18], obtaining satisfactory results. The use of a smartphone or HoloLens does not significantly modify the surgical procedure neither requires additional installation of devices or tools inside the OR, unlike other systems such as CAS with an optical tracking system.

## 5. Conclusions

In this study, we have presented a guiding tool for PSIs placement based on AR. The system has been validated in a phantom designed to provide a more realistic setup than conventional ones. The results obtained from this study are promising and demonstrate that using AR for guidance can significantly reduce the risk of high placement errors and ensures an accurate installation close to the target. We believe that the presented system can overcome the present limitations of PSIs—the impossibility of verifying their placement objectively—conveniently and effectively.

## Figures and Tables

**Table 1:** Descriptive statistics of maximum osteotomy deviations for each phantom. Q25 and Q75 represent the 25th and 75th percentile. Mdn represents the median.

		Freehand			Smartphone			HoloLens		
		C	S	Total	C	S	Total	C	S	Total
Conventional phantom (no silicone)	Min	0.27	0.47	0.27	0.28	0.38	0.28	0.45	0.34	0.34
	Q25	1.02	0.96	1.01	0.78	0.77	0.77	0.83	0.83	0.83
	Mdn	1.48	1.81	1.70	1.15	0.95	1.04	1.12	1.01	1.06
	Q75	2.64	2.56	2.56	1.38	1.42	1.42	1.60	1.39	1.54
	Max	7.27	3.45	7.27	2.20	2.52	2.52	2.87	3.00	3.00
Realistic phantom (silicone)	Min	0.79	0.41	0.41	0.33	0.54	0.33	0.51	0.92	0.51
	Q25	2.37	2.09	2.10	0.94	1.36	1.07	1.01	1.66	1.13
	Mdn	3.70	3.24	3.37	1.22	2.21	1.54	1.40	2.50	1.84
	Q75	10.02	4.13	6.13	2.12	3.01	2.55	2.04	2.89	2.68
	Max	54.03	9.83	54.03	5.00	4.89	5.00	3.89	4.13	4.13

## Data Availability

Data is contained within the article.

## Supplementary Materials

The following are available online at https://www.mdpi.com/article/10.3390/s21237824/s1, Video S1: AR guidance with smartphone, Video S2: AR guidance with HoloLens 2, Table S1: Details about the hardware and software used for the study.
