# Supplementary material for: Augmented Reality as a Tool to Guide PSI Placement in Pelvic Tumor Resections
Source: Sensors (Basel). 2021 Nov 24;21(23):7824. doi: 10.3390/s21237824 (PMC8659846; doi:10.3390/s21237824)
Supplement: Supplementary file 1 [file sensors-21-07824-s001.zip › sensors-1471489-SupplementaryMaterial/sensors-1471489-Table S1.pdf]

sensors

MDPI

Article

Augmented Reality as a Tool to Guide PSI Placement in Pelvic Tumor Resections

Mónica García-Sevilla [1,2], Rafael Moreta-Martinez [1,2], David García-Mato [1,2], Alicia Pose-Diez-de-la-Lastra [1,2], Rubén Pérez-Mañanes [2,3], José Antonio Calvo-Haro [2,3] and Javier Pascau [1,2,*]

[1] Departamento de Bioingeniería e Ingeniería Aeroespacial, Universidad Carlos III de Madrid, 28911 Leganés, Spain; mongarci@pa.uc3m.es (M.G.-S.); rmoreta@pa.uc3m.es (R.M.-M.); dgmato@ing.uc3m.es (D.G.-M.); apose@ing.uc3m.es (A.P.-D.-d.-l.-L.)
[2] Instituto de Investigación Sanitaria Gregorio Marañón, 28007 Madrid, Spain; rubenperez.phd@gmail.com (R.P.-M.); calvoharo@yahoo.es (J.A.C.-H.)
[3] Servicio de Cirugía Ortopédica y Traumatología, Hospital General Universitario Gregorio Marañón, 28007 Madrid, Spain
* Correspondence: jpascau@ing.uc3m.es; Tel.: +34-91-624-8196

Supplementary Materials:

Table S1. Details about the hardware and software used for the study .

HARDWARE/ SOFTWARE	DEVICE OR PROGRAM	COMPANY	PURPOSE
HARDWARE	Google Pixel 4 XL	Google Inc., CA, USA	AR guidance with a smartphone
HARDWARE	HoloLens 2	Microsoft, Redmond, WA, USA	AR guidance with a head-mounted display
HARDWARE	Ultimaker 3 extended 3D printer	Ultimaker B.V., Netherlands	3D printing of the bone phantom, the PSIs, and the reference frame
HARDWARE	Polaris Spectra optical tracking system	NDI, Waterloo, Canada	Navigation for the assessment of PSIs placement
SOFTWARE	Unity platform	Unity Software Inc., San Francisco, CA, USA	Build the AR apps
SOFTWARE	Vuforia development kit	Parametric Technology Corporation Inc., Boston, MA, USA	Pattern recognition of the AR marker
SOFTWARE	3D Slicer	Free, open-source software (BSD-style license) with no associated company	Navigation for the assessment of PSIs placement

SOFTWARE	PLUS	Free, open-source library. Supported by the Plus Toolkit Community and PerkLab, Queen's University from 2011 to 2017	Communication between the optical tracker (Polaris) and the navigation software (3D Slicer)
SOFTWARE	Meshmixer	Autodesk, Inc., USA	Design of PSIs and phantom
SOFTWARE	Cura	Ultimaker B.V., Netherlands	Generation of the g-codes for 3D printing
